# Bioremediation of Aflatoxin B_1_ by *Meyerozyma guilliermondii* AF01 in Peanut Meal via Solid-State Fermentation

**DOI:** 10.3390/toxins16070305

**Published:** 2024-07-04

**Authors:** Wan Zhang, Changpo Sun, Wei Wang, Zhongjie Zhang

**Affiliations:** 1 College of Engineering, China Agricultural University, No. 17 Tsinghua East Road, Beijing 100083, China; zwan@ags.ac.cn; 2 Academy of National Food and Strategic Reserves Administration, Beijing 100037, China; chpsun@163.com

**Keywords:** single factor, response surface methodology, shallow pans, fermentation bags, nutrient composition

## Abstract

The use of microorganisms to manage aflatoxin contamination is a gentle and effective approach. The aim of this study was to test the removal of AFB_1_ from AFB_1_-contaminated peanut meal by a strain of *Meyerozyma guilliermondii* AF01 screened by the authors and to optimize the conditions of the biocontrol. A regression model with the removal ratio of AFB_1_ as the response value was established by means of single-factor and response surface experiments. It was determined that the optimal conditions for the removal of AFB_1_ from peanut meal by AF01 were 75 h at 29 °C under the natural pH, with an inoculum of 5.5%; the removal ratio of AFB_1_ reached 69.31%. The results of simulating solid-state fermentation in production using shallow pans and fermentation bags showed that the removal ratio of AFB_1_ was 68.85% and 70.31% in the scaled-up experiments, respectively. This indicated that AF01 had strong adaptability to the environment with facultative anaerobic fermentation detoxification ability. The removal ratio of AFB_1_ showed a positive correlation with the growth of AF01, and there were no significant changes in the appearance and quality of the peanut meal after fermentation. This indicated that AF01 had the potential to be used in practical production.

## 1. Introduction

Peanut meal is a byproduct of peanut oil extraction that is rich in nutrients, especially protein content (exceeding 40%). It can be used for brewing soy sauce or as feed [1]. However, the contamination of peanuts or peanut meal itself by mycotoxins during storage and processing, especially aflatoxins, greatly reduces its application value [2]. Aflatoxins are secondary metabolites produced by *Aspergillus* species such as *A. flavus* and *A. parasiticus*, mainly composed of a difuran ring and coumarin. More than 20 derivatives with similar structures have been discovered, with the most common being aflatoxins B_1_ (AFB_1_), B_2_, G_1_, G_2_, M_1_ and M_2_ [3]. Among them, AFB_1_ has the strongest toxicity with carcinogenicity [4], genotoxicity [5], neurotoxicity [6] and cytotoxicity [7].

There are physical, chemical and biological methods for removing AFB_1_ from peanut meal [8,9]. The biological method has a relatively mild effect and does not damage the nutrients of the peanut meal, attracting widespread attention from researchers. This method mainly removes AFB_1_ from peanut meal through microbial fermentation, and its mechanisms of action are adsorption and degradation. Adsorption refers to the use of microbial cell walls or extracellular secretions to transfer AFB_1_ [10]. This is mainly achieved through the formation of non-covalent bonds between polysaccharides on microbial cell walls, such as β-glucan, and AFB_1_ molecules, preventing their release in the gastrointestinal tract and allowing them to be excreted from the body with feces [11]. The yeast cell wall added to feed currently reduces the damage of AFB_1_ to animals through adsorption [12,13]. Degradation refers to the conversion of AFB_1_ into non-toxic or low-toxicity substances through the growth processes of the microorganisms themselves [14]. This is mainly because microorganisms can produce active substances such as proteins or non-protein components under external stimuli. These substances have specificity and can target the toxic functional groups of AFB_1_, breaking them down into smaller molecules or changing their functional group structure to reduce or disappear their toxicity, achieving the goal of reducing the harm of AFB_1_ [15,16].

The clearance effect of different microorganisms on AFB_1_ varies. Some can remove AFB_1_ from aqueous solutions but have no effect on AFB_1_ in complex materials. This is due to the growth characteristics of the microorganisms themselves and their specificity in AFB_1_ metabolism [17]. However, actual detoxification scenarios often involve complex materials, including food and feed. Therefore, mining microorganisms that can effectively remove AFB_1_ from complex materials is of great significance for production applications. In recent years, microorganisms that can remove AFB_1_ from food or feed have been reported. Zhang et al. used *Lactobacillus helveticus* FAM22155 to remove AFB_1_ in wheat bran in different ways. After 48 h, it was found that the live cells of the strain achieved the highest removal ratio of AFB_1_ through solid-state fermentation (88.6%). Meanwhile, the protein extracted from the fermented bran of this strain acted on the wheat bran with a removal ratio of 85.3%. The intracellular extract from the fermentation broth of the strain gained a removal ratio of 36.3%. The remaining components had almost no degradation. This indicated that *L. helveticus* FAM22155 mainly degraded AFB_1_ through intracellular enzymes, and solid-state fermentation was currently the optimal method [14]. However, the removal efficiency of AFB_1_ may vary depending on the environmental conditions of solid-state fermentation. Escriva et al. used *L. plantarum* B3 and *L. Paracasei* B10 to remove AFB_1_ from dough and bread with and without yeast, respectively. After 24 h of fermentation, the removal ratios of AFB_1_ by *L. plantarum* B3 in yeast and yeast-free dough were 43.8% and 27.1%, respectively. The removal ratios in bread were 51.8% and 55.0%, respectively. Meanwhile, the removal ratios of AFB_1_ from the dough by *L. paracasei* B10 were 11.0% and 26.7%, respectively. The removal ratios in bread were 10.6% and 30.6%, respectively. The results showed that the presence of yeast had a promoting effect on the removal of AFB_1_ by *L. Plantarum* B3, while it had the opposite effect on *L. Paracasei* B10. Thus, the two strains had different removal ratios of AFB_1_ in dough and bread, indicating that substrates and reaction conditions, namely the environment, had a significant impact on the microbial removal of AFB_1_ [18]. Therefore, optimizing the solid-state fermentation conditions is of great significance for improving the biological control effect of AFB_1_.

Optimizing solid-state fermentation conditions can also improve the microbial removal rate of AFB_1_. For instance, Li et al. utilized two strains of bacteria, *Bacillus velezensis* and *Pediococcus acidilactici*, which not only effectively removed AFB_1_ from peanut meal but also improved the substrate quality [19]. In 2024, Luo et al. optimized the conditions for *Trametes versicolor* to degrade AFB_1_ in corn through fermentation using the response surface methodology, increasing the degradation ratio of AFB_1_ by 25% [9]. Due to the different characteristics of microorganisms, the required fermentation conditions are also different. Optimizing solid-state fermentation conditions can maximize the detoxification potential of the microorganism and reduce the possibility of misjudgment. Moreover, solid-state fermentation is a process in which microorganisms consume energy and produce excessive carbon dioxide. If the microorganism has facultative anaerobic characteristics, it may ferment under anaerobic conditions to remove AFB_1_ so as to reduce energy loss and reduce carbon emissions. This will greatly promote the process of large-scale application of microbial detoxification of AFB_1_.

In the authors’ previous research, a strain of *Meyerozyma guilliermondii* (AF01) with facultative anaerobic ability was screened. The strain was proven to be capable of removing AFB_1_ in vitro, including adsorption and degradation [20]. To test whether the strain has a detoxification effect on AFB_1_ in actual materials, an in vivo detoxification experiment is designed in this study. Its ability to remove AFB_1_ from peanut meal through solid-state fermentation was tested. In addition, to optimize the detoxification conditions, single-factor and response surface experiments were conducted. The theoretical optimal detoxification conditions obtained were used for large-scale fermentation using two fermentation methods, shallow plate and fermentation bag, to test the strain’s ability to remove AFB_1_ under aerobic and anaerobic conditions. To comprehensively evaluate the production and application potential of this strain, the impact of its fermentation on the quality of peanut meal was also tested. This is the first report on the removal of AFB_1_ in food or feed by *M. guilliermondii*, which can provide new microbial resources and detoxification technology support for the biological control of AFB_1_.

## 2. Results and Discussion

### 2.1. Analysis of Single-Factor Experimental Results

**Figure 1:**
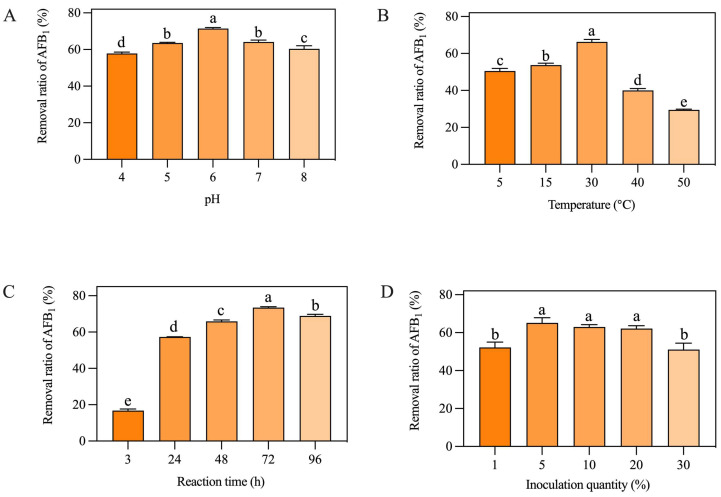
Effects of different factors on the removal of aflatoxin B_1_ (AFB_1_) from peanut meal by the AF01 strain: (**A**) pH; (**B**) temperature; (**C**) reaction time; (**D**) inoculation dose. Different lowercase letters on the same coordinate axis indicate significant differences (*p* < 0.05), while the same lowercase letters on the same coordinate axis indicate insignificant differences (*p* > 0.05) according to Duncan’s multiple range test. To test the growth of the AF01 strain in highly toxic peanut meal, and to investigate the effect of strain growth on AFB_1_ removal, the strain density was tested in different inoculation groups of peanut meal. The results of plate dilution coating are shown in Table 1. After 72 h of cultivation, except for the 30% inoculation group, the colony density of the AF01 strain in all other reaction systems increased by two orders of magnitude. The results show that the AF01 strain can achieve rapid proliferation by introducing secondary seed liquid microbial agents into peanut meal, indicating that it can utilize the nutrients in peanut meal to complete its own growth and reproduction. In the later stages, the inoculation method can be optimized to increase the growth density of the AF01 strain and prolong the action time of biocontrol strains [21]. When the inoculation dose was 5%, the final colony density of the AF01 strain in peanut meal was the highest, at 6.5 × 10^9^ CFU/g. The colony densities at different times were as shown in Figure 2 and Figure 3. A single colony of AF01 strain appeared as white scattered small dots on PDA plates, which can be counted clearly. The changes in colony density on plates with different dilutions can intuitively reflect the growth trend of AF01 in peanut meal.

As shown in Figure 1A, the removal ratio of AFB_1_ from peanut meal was significantly affected by the pH value. When the pH was 6.0, the AF01 strain had the highest removal rate. When the pH value was 4.0, the removal ratio of the AF01 strain was the lowest. When the pH was 7.0, the AF01 strain had a slightly lower removal ratio of AFB_1_ than when the pH was 6.0. Figure 1B shows the removal ratio of AFB_1_ by the AF01 strain at different cultivation temperatures. When the cultivation temperature was 30 °C, the AF01 strain had the highest removal ratio of AFB_1_. When the temperature increased to 40 °C and 50 °C, the removal ratio of AFB_1_ significantly decreased. Figure 1C shows that the removal ratio of AFB_1_ from peanut meal by the AF01 strain was time-dependent, but there was no significant increase in the removal ratio of AFB_1_ after 72 h. As shown in Figure 1D, the inoculation dose of the AF01 strain had a significant impact on the removal ratio of AFB_1_. When the inoculation dose was 1% or 30%, the removal ratio of AFB_1_ was significantly lower than in the other groups. When the inoculation dose was 5%, the AF01 strain had the highest removal ratio of AFB_1_. Overall, changes in reaction time and temperature had a significant impact on the removal rate of AFB_1_, followed by inoculation quantity and pH of the substrate. Therefore, temperature and reaction time were selected as the two dependent variables for the response surface experiment. The natural pH of peanut meal used in this study was 5.6, which was close to its optimal reaction pH of 6. Therefore, considering the operability of the subsequent experiments and the convenience in actual production, inoculation quantity was chosen as the third dependent variable in the following response surface experiment.

The removal ratio of AFB_1_ from peanut meal showed that the treatment group with an inoculation dose of 5.5% also had the highest removal ratio of AFB_1_, which was 65.19%. In addition, the results showed that the growth rate of the AF01 strain was the fastest within 24 h, with a growth ratio of two orders of magnitude, and then tended to stabilize. Correspondingly, after 24 h of cultivation, the removal ratio of AFB_1_ by the AF01 strain rapidly increased from 41.51% after 3 h to 82.09%, indicating that this strain will rapidly decompose AFB_1_ during the growth process. This phenomenon was consistent with the findings of Ma et al. [22]: by fitting the detoxification curve and growth curve of T-2 toxin-degrading bacteria, it was found that when the strain was in a logarithmic growth period, T-2 toxin was rapidly consumed and the degradation ratio also increased exponentially, indicating that the degrading bacteria can use toxins as a carbon source for growth and reproduction. The enzyme formation and cell growth of a filamentous fungus that can produce cellulase, discovered by Jia et al. [23], exhibited a coupling type, meaning that the cells utilized substrates at a faster rate during the logarithmic growth phase, which is consistent with the results of this study.

**Figure 3:**
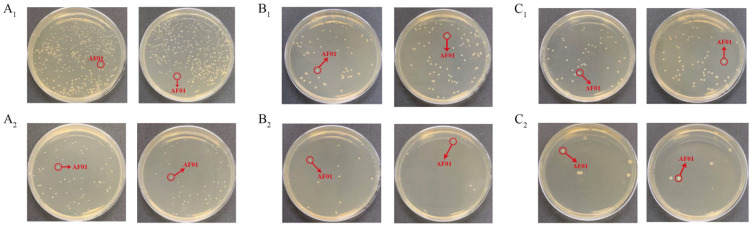
The density of the AF01 strain in peanut meal after 24 h, 48 h and 72 h of reaction: (**A_1_**) 24 h, 10^−6^ dilution; (**A_2_**) 24 h, 10^−7^ dilution; (**B_1_**) 48 h, 10^−7^ dilution; (**B_2_**) 48 h, 10^−8^ dilution; (**C_1_**) 72 h, 10^−7^ dilution; (**C_2_**) 72 h, 10^−8^ dilution. The circled and similarly shaped single colony is the strain of AF01.

In the preliminary research of our laboratory, the mechanism of AFB_1_ removal by AF01 strain was elucidated [20]. That is, the AF01 strain can both degrade and adsorb AFB_1_. The main components of degrading AFB_1_ are living cells and intracellular enzymes produced by living cells. The main adsorbent for AFB_1_ is the fermentation supernatant of AF01 strain, which is irreversible. The results of the current study indicated that the removal rate of AFB_1_ from peanut meal by AF01 strain increased with the extension of fermentation time (Figure 1). At the same time, the density of AF01 strain in peanut meal also showed an upward trend (Figure 3). When the density of the AF01 strain significantly increased, the removal rate of AFB_1_ also significantly increased at the corresponding time. This indicated a certain correlation between the two. This discovery is also consistent with the findings of Lou et al. [9] in 2023. They used *T. versicolor* to remove AFB_1_ from corn through solid-state fermentation and found that the removal rate of AFB_1_ was closely related to the growth of *T. versicolor*. Furthermore, the growth of *T. versicolor* enhanced the removal rate of AFB_1_. Therefore, based on the analysis of the mechanism of the removal of AFB_1_ by AF01 in the early stage and in the peanut meal fermentation experiment, the removal rate of AF01 will be enhanced with the growth and reproduction of the living cells of AF01.

In our previous research, we identified the degradation product of AFB_1_ by AF01 as aflatoxicol (AFL). Its toxicity is less than that of AFB_1_ [24,25]. The conversion of AFB_1_ to AFL is considered a protective mechanism that can prevent the formation of AFBO in animals, which is well known to attack DNA and induce genetic toxicity in the body [26]. In this experiment, some AFL was also extracted from peanut meal fermented by AF01 strain through UPLC-MS detection, but the amount was relatively small. This may be because the immunoaffinity column used for extraction is only specific to AFB_1_, so only a small amount of AFL can be bound. Overall, the AF01 strain has a dual effect of adsorption and degradation in the removal of AFB_1_ from peanut meal, which is an advantage of the AF01 strain compared to other biocontrol strains.

### 2.2. Analysis of Response Surface Experiment Results

Based on the results of the single-factor experiment, time (*A*), inoculation quantity (*B*) and temperature (*C*) with significant impact were selected to conduct a 3-factor and 3-level Design Expert 8.0.6.1 response surface experiment design. The removal ratio of AFB_1_ (*Y*) was used as the response value, and the optimal conditions for the fermentation of *M. guilliermondii* AF01 strain to remove AFB_1_ from peanut meal were screened. The Box–Behnken experimental design and results are shown in Table 2, and the analysis of variance is shown in Table 3. As shown in Table 2, the removal ratio of AFB_1_ by AF01 strain obtained under different fermentation conditions of time, inoculation quantity and temperature varied.

According to Table 3, the *p*-value of the model was less than 0.0001, so the model was extremely significant. The *p*-value of the lack of fit term was 0.1712, which was larger than 0.05, indicating that the lack of fit term was not significant. These two indicators indicated that the regression equation had a good fitting effect and that the experimental design scheme was operable. The determination coefficient R^2^ of the model (R^2^) was 0.9907 and the adjusted determination coefficient R^2^ (R^2^_Adj_) was 0.9788, indicating that the model was reasonable and feasible. The experimental error was relatively small, enabling it to better reflect the actual relationship between the removal of AFB_1_ from peanut meal and its response value [27]. Therefore, this model can be used for analyzing and predicting the optimization of AFB_1_ removal conditions in complex matrices. According to the *p*-value, the quadratic terms *A*^2^, *B*^2^ and *C*^2^ had an extremely significant impact on the results (*p* < 0.0001), indicating that the impact of each factor on the removal ratio of AFB_1_ was not generally linear. The impact of terms *A* and *C* on the results was highly significant (*p* < 0.01). The interaction of three factors had a significant impact on the results (*p* < 0.05). Based on the *F* value, the order of influence of the three factors on the removal rate of AFB_1_ was *C* (temperature) > *A* (time) > *B* (inoculation quantity). This was similar to the findings of Yu et al. on the removal of ZEN from DDGS, where action time and temperature were key factors affecting the toxin removal rate [28].

Through the analysis of Design-Expert 8.0.6.1 software, the equation of the multivariate quadratic response surface regression model was obtained as follows:

(1) $$Y=-189.93769+2.95723A+4.32339B+9.34781C-0.020301AB\;-0.010625AC+0.053444BC-0.016881A^{2}-0.39498B^{2}-0.15241C^{2}$$

where *A* is the action time (h), *B* is the inoculation quantity (%) and *C* represents the temperature (°C).

**Figure 4:**
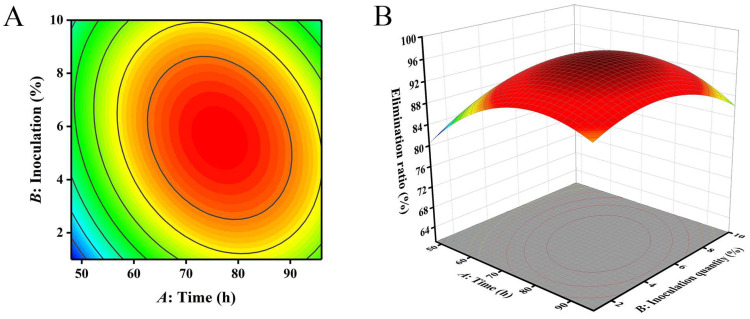
The influence of reaction time and inoculation quantity on the removal ratio of aflatoxin B_1_ (AFB_1_): (**A**) contour; (**B**) response surface.

**Figure 5:**
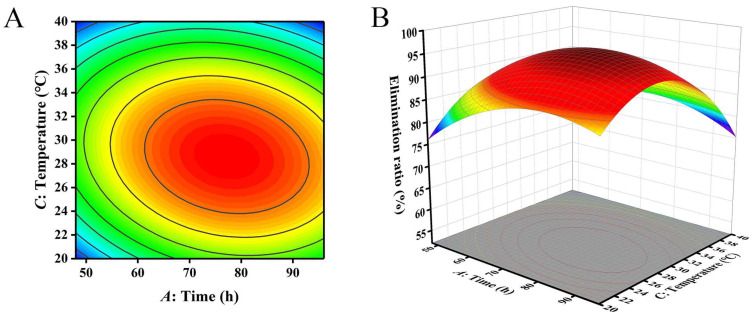
The influence of reaction time and temperature on the removal ratio of aflatoxin B_1_ (AFB_1_): (**A**) contour; (**B**) response surface.

**Figure 6:**
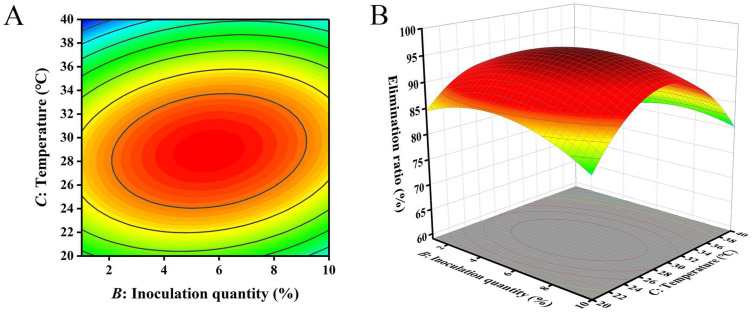
The influence of inoculation quantity and temperature on the removal ratio of aflatoxin B_1_ (AFB_1_): (**A**) contour; (**B**) response surface.

The closer the contour line is to a circular or saddle shape, the less significant the interaction between the two factors. The closer the shape is to an ellipse, the more significant the interaction becomes [29,30]. The response surface curves of the three factors all exhibited a parabolic shape, with the opening downward, indicating the presence of a peak. The steeper the slope of the response surface, the more significant the interaction between various factors. The influence of various factors on the contour lines and response surfaces of the results is shown in Figure 4, Figure 5 and Figure 6. From Figure 4, Figure 5 and Figure 6, it can be seen that the interaction of various factors had a significant impact on the results (*p* < 0.05). The contour lines of time and temperature, inoculation quantity and temperature were elliptical, indicating that the interaction between the two factors had a significant impact on the results. The contour lines of time and inoculation quantity were relatively gentle, indicating that the interaction between the two factors had less impact on the results than the other two groups. This result is consistent with the results in Table 3.

The optimal conditions for removing AFB_1_ from peanut meal were obtained using Design Expert 8.0.6.1 software: action time of 75.17 h, inoculation dose of 5.50% and temperature of 29.01 °C. The theoretical maximum removal ratio of AFB_1_ under these reaction conditions was 68.68%. To test the accuracy of the model’s prediction, three repeated experiments were conducted with an action time of 75 h, an inoculation dose of 5.5% and a temperature of 29 °C. The removal ratio of AFB_1_ was found to be 69.31%, which was close to the predicted value. Therefore, it is feasible to use response surface analysis to establish parameters for the removal of AFB_1_ from peanut meal.

### 2.3. Analysis of the Removal Effect of Aflatoxins in Simulated Actual Production

Two methods of applying fermentation strains in actual production were adopted: shallow-plate fermentation and fermentation-bag fermentation. The shallow-plate fermentation process is shown in Figure S1, where the growth of bacteria can be observed in the fermented peanut meal. Due to the loss of moisture, the surface of the peanut meal cracked and was not as smooth as that before fermentation. Therefore, it was necessary to regularly spray sterile water upwards during the reaction process to keep the peanut meal moist.

**Figure 7:**
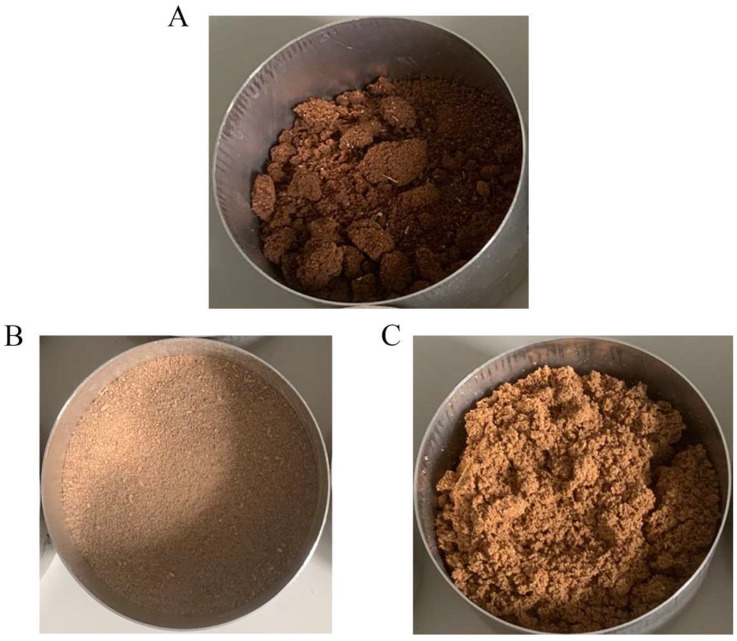
Comparison chart of peanut meal before and after fermentation: (**A**) before fermentation; (**B**) after shallow-plate fermentation; (**C**) after fermentation in the fermentation bag.

However, after fermentation, the texture of the peanut meal was less hard and still loose. As shown in Figure 7, there was no significant change in the color of the peanut meal before and after fermentation, and there was no adhesion phenomenon. This is also the advantage of yeast fermentation compared to mold fermentation—that is, yeast fermentation does not have adverse effects on the appearance of the substrate [31]. After 75 h of reaction, the removal ratio of AFB_1_ from peanut meal in the shallow plates was 68.85%, indicating that the AF01 strain can effectively remove AFB_1_ from peanut meal in shallow-plate fermentation.

The process of AFB_1_ removal by the AF01 strain in the fermentation bags is shown in Figure S2. During the fermentation process, the AF01 strain continuously produced carbon dioxide due to its own growth and reproduction, resulting in a swollen fermentation bag. From another perspective, this also indicates that the AF01 strain grew well in the fermentation bag [32,33].

As shown in Figure 7 and Figure S2, the fermented peanut meal had no significant change in color. Through touch and smell, it could be found that the fermented peanut meal had a softer texture and a special wine aroma. The solid-state fermentation of the AF01 strain had no adverse effects on the appearance of the peanut meal and gave it a unique flavor. Based on the sensitivity of animals to odor, this could also increase their food intake and promote their digestion and absorption of feed [34]. In addition, anaerobic conditions can prevent the growth of most harmful bacteria, reduce the probability of peanut meal spoilage, and help improve the quality of the peanut meal [35]. After 75 h of reaction, the removal ratio of AFB_1_ from peanut meal in the fermentation bag was 70.31%, indicating that the actual fermentation-bag fermentation mode can still effectively remove AFB_1_ from peanut meal in production. The removal ratio of AFB_1_ by *M. guilliermondii* obtained is superior to the efficiency previously reported. Li et al. used *Bacillus velezensis* LB-Y-1 and *P. acidilactici* LC-9-1 for two-stage solid-state fermentation, gaining a 43.68% removal ratio after 72 h. The AFB_1_ content in the substrate (38.37 μg/kg) was lower than that in this study (98.87 μg/kg) and the operation was not as simple as in this experiment [19]. Lou et al. used *T. versicolor* to remove AFB_1_ from corn through solid-state fermentation. The removal ratio of AFB_1_ after 5 days was 65% [9].

In summary, as a facultative anaerobic microorganism, the AF01 strain had the ability to remove AFB_1_ from complex materials in both shallow-plate and fermentation-bag fermentation, and it had no adverse effects on the appearance or color of the fermentation substrate, while also endowing the substrate with a special flavor, improving its commercial value as feed. Therefore, the AF01 strain has the potential for practical applications in production.

### 2.4. The Effect of AF01 Strain Fermentation on the Nutrition of Peanut Meal

The nutritional indicators of peanut meal before and after detoxification were tested. As shown in Table 4, the total sugar content and total amino acid content both increased after fermentation, while the phytic acid content decreased.

As shown in Figure S3, the R^2^ of the standard curve for phytic acid determination was 0.9991, indicating a good linear relationship. After calculation, the phytic acid content in the peanut meal was 1.47% before fermentation. After fermentation with the AF01 strain, the phytic acid content in the peanut meal was 0.68%, a decrease of 0.79%. As shown in Figure S4, the R^2^ of the standard curve for glucose mass concentration was 0.9968, indicating a good linear correlation. The results showed that the total sugar content in the control peanut meal was 35.27%. After fermentation by the AF01 strain, the total sugar content in the peanut meal was 39.37%, an increase of 4.10%.

The determination of the amino acid standard solution was repeated three times, and the obtained spectrum is shown in Figure S5. The detection results of amino acid content in peanut meal before and after fermentation showed that the peanut meal contained rich amino acid components (Table 5). The highest contents in peanut meal were of glutamic acid, aspartic acid and arginine. Arginine is a crucial amino acid in the process of microbial synthase, which provides favorable conditions for the growth of strains in peanut meal [36]. After fermentation treatment with the AF01 strain, the amino acid composition in the peanut meal remained essentially unchanged, but the concentration of each amino acid increased, indicating that the fermentation with the AF01 strain improved the quality of the peanut meal.

In the study, the total amino acid content in fermented peanut meal increased by 4.39%, which was consistent with the general law of solid-state fermentation. Numerous studies showed that the increase in amino acids in substrates such as peanut meal, soybean meal, or corn after solid-state fermentation was largely related to the microorganisms used in the fermentation [37]. Cui et al. used *Trichoderma koningii* and *A. niger* for solid-state fermentation in tea residue, resulting in a 3% and 5% increase in total hydrolyzed amino acids, respectively [38]. Shi et al. used *A. niger*, *Candida utilitis* and *Bacillus subtilis* for solid-state fermentation in drumstick tree substrate, resulting in a 3.57% increase in amino acids [37]. However, not all microorganisms increased the amino acid content of the substrate through solid-state fermentation. Suprayogi et al. used *A. oryzae* for solid-state fermentation in soybean meals and found no significant change in amino acid content. The reason may be related to the poor protease activity, secretion ability and adaptability to the environment of *A. oryzae* during the fermentation process [39]. It is also possible that the germination of *A. oryzae* spores may consume some essential amino acids [40]. *A. oryzae* is also a type of microorganism reported to have the ability to degrade AFB_1_. Therefore, the total performance of *M. guilliermondii* used in this study is relatively strong.

The total sugar content in fermented peanut meal also increased by 4.10%. There are two main reasons why microorganisms can increase the total sugar and amino acid content in substrates through solid-state fermentation. One reason is that microorganisms produce various enzymes, including proteases and hydrolases, which can convert large molecules into smaller molecules that are more absorbed by animals or humans, such as sugars and amino acids [39]. Conversely, it may be due to some biological pathways of microorganisms themselves that produce these substances. For example, an increase in pyruvate and fermentation intermediates such as 2-oxoglutarate produces lysine [41]. In the process of glycolysis, serine is synthesized by upregulating the glycolytic pathway. This is consistent with the results obtained in this study. Moreover, *M. guilliermondii* has the characteristic of facultative anaerobic fermentation, with more diverse fermentation methods and richer metabolic pathways. This is also an advantage of using *M. guilliermondii* for detoxification through fermentation [42,43].

The nutritional components of fermented peanut meal, such as total sugar and amino acid contents, were improved, indicating that the AF01 strain can improve the nutritional composition of peanut meal through fermentation. Based on the results, it was inferred that the AF01 strain may secrete various extracellular proteins during its growth process, improving the substrate composition and making it more suitable for use as feed [44]. This was similar to the findings of Li et al., who used various probiotics to remove AFB_1_ from peanut meal through solid-state fermentation, improving the quality of the peanut meal in the process [19]. Microbial fermentation and degradation of fungal toxins in feed can not only reduce the mycotoxins to the national standard limit level but also improve the nutritional content of the feed, which has great production and application prospects.

Overall, *M. guilliermondii* AF01 exhibits both adsorption and degradation abilities towards AFB_1_, demonstrating strong detoxification ability in aqueous solutions and complex materials. This strain also has facultative anaerobic properties and can adapt well to different environments, resulting in great potential for application in production. This is also the reason why this strain was chosen for in-depth research. The safety of strains is also an important factor to consider for long-term use. *M. guilliermondii* has been studied for over 40 years and has an inhibitory effect on the growth of pathogenic bacteria. It has been used for disease management of fruit trees or postpartum fruits [45]. Acute toxicity tests with mice demonstrated that it is not harmful to animal bodies [46]. The toxicity of the degradation products of AFB_1_ by this strain was greatly reduced. However, although *M. guilliermondii* is distributed in the environment, food and gut [47], more comprehensive toxicological experiments are needed to evaluate its application security.

## 3. Conclusions

The results of this study indicated that *M. guilliermondii* (AF01) could grow and reproduce well in peanut meal, whether under aerobic or anaerobic conditions, laying the foundation for its detoxification of AFB_1_ in complex materials. AF01 had a strong removal effect on AFB_1_ in peanut meal, which exceeded 70% within 3 days of fermentation. Moreover, whether in shaking flasks or large-scale fermentation systems, AF01 demonstrated good AFB_1_ removal ability, indicating that it was less affected by the environment and could be suitable for detoxification in different scenarios. After fermentation, the sensory and nutritional quality of the peanut meal was improved, further demonstrating the potential application of AF01 in actual production. However, more comprehensive toxicological experiments are needed to evaluate its safety. In summary, this study provided a new and promising microbial resource for the biocontrol of AFB_1_. Meanwhile, the solid-state fermentation technology used in this study could effectively achieve the removal of AFB_1_ from complex materials, providing a reference for other microorganisms to remove mycotoxins from food or feed through solid-state fermentation.

## 4. Materials and Methods

### 4.1. Chemicals and Regents

Sulfosalicylic acid, sodium citrate and the amino acid mixed standard were purchased from Solarbio (Beijing, China). The commercial standard of AFB_1_ was purchased from Pribolab, Ltd. (Qingdao, China). Tryptone and yeast extracts were purchased from Oxoid, Ltd. (Basingstoke, UK). Potato extract, glucose and agar were provided by Aoboxing Co. (Beijing, China). Kanamycin was provided by Genview (Chelmsford, CA, USA). High-performance liquid chromatography (HPLC)-grade methanol was obtained from Thermo Fisher Scientific (Waltham, MA, USA). Na_2_HPO_4_, KH_2_PO_4_, NaCl, NaOH, H_2_SO_4_ and HCl were purchased from National Pharmaceutical Group Chemical Reagent Co., Ltd. (Beijing, China). Phenol and CuSO_4_ were purchased from Macklin (Shanghai, China). The AFB_1_ immunoaffinity column was purchased from Hua’an Maike Biotechnology Co., Ltd. (Beijing, China). Isopropyl-β-D-thiogalactopyranoside (IPTG) was purchased from Boao Tuoda Technology Co., Ltd. (Beijing, China). KCl and Na_2_SO_4_ were purchased from Beijing Chemical Plant (Beijing, China). Sodium phytate was purchased from Yuanye Biotechnology Co., Ltd. (Shanghai, China). K_2_SO_4_ was purchased from Xilong Chemical Co., Ltd. (Yulin, China). The Agilent Poroshell 120 EC-C18 chromatography column was purchased from Waters Company (Milford, MA, USA). Sterile disposable applicator sticks were purchased from Changde Beekman Biotechnology Co. (Changde, China). Qualitative filter papers were purchased from Hangzhou Fuyang Beimu Pulp & Paper Co., Ltd. (Hangzhou, China). Microfiber filter papers were purchased from Whatman (Maidstone, UK). Deionized water was produced using the Elix 20 water purifier (Millipore, MA, USA).

### 4.2. Preparation of AF01 Strain Seed Solution

The glycerol bacteria were removed from a −80 °C refrigerator and streaked onto potato dextrose agar (PDA) for 48 h of cultivation. Then, a single bacterium was selected and cultured in potato dextrose broth (PDB) for 24 h until the logarithmic phase. Next, 5 mL of seed solution was taken and transferred to 4 bottles of new 100 mL PDB medium. Finally, the bottles were shaken and cultured for 36 h until the OD_600_ was 40, which was the maximum OD. At this time, the AF01 strain was in the late logarithmic growth stage.

### 4.3. Peanut Meal Preparation

The pH value of the peanut meal was around 5.6, and the concentration of AFB_1_ in its dry state was 98.87 μg/kg. First, 20 g of peanut meal was weighed into a 300 mL triangular flask and sterilized using a Hirayama autoclave (Pingshan, Tokyo, Japan) at 121 °C for 20 min. Water was added to the Elix 20 water purifier (Millipore, MA, USA) to obtain deionized water. Then, deionized water was transferred to a triangular flask and heated using a Hirayama autoclave (Pingshan, Tokyo, Japan) at 121 °C for 20 min to obtain the sterile water. After cooling to room temperature, 8 g of sterile water was added to the triangular flasks containing peanut meal on an ultraclean workbench. The mixture was stirred well using a sterile disposable applicator stick by hand; then, the seed liquid of the AF01 strain was added.

### 4.4. AFB_1_ Detection Method

Ultrahigh-performance liquid chromatography–mass spectrometry (UPLC-MS) was used to monitor the AFB_1_ residue in the sample. The chromatographic column used was a 2.1 mm × 100 mm Agilent Poroshell 120 EC-C18 column with a pore size of 2.7 μm. The injection volume was 2 μL. The flow rate was 0.2 mL/min, and the temperature was set to 30 °C. The mobile phase was gradient elution, which was divided into an aqueous solution containing 0.1% formic acid and 5 mM ammonium formate, and a methanol solution containing 0.1% formic acid. A 10% methanol solution was maintained from 0 min to 1 min. Next, the methanol solution was gradually increased to 45% from 1 min to 1.5 min, and then it was gradually increased to 100% from 1.5 min to 8.5 min, where it remained for 1 min. Finally, it dropped back to 10% between 9.5 min and 10 min.

An Agilent 6545 ESI Q-TOF mass spectrometer was used for the mass spectroscopy measurements. The ionization source was the positive source of electric spray (ESI^+^), the nebulizer was 40 psig and the capillary voltage was 4.0 kV. The fragmentor voltage was 175 V, the skimmer voltage was 65 V, and the gas temperature was 300 ℃. The auxiliary gas (N_2_) flow rate was 5 L/min. The temperature of the gas inside the sheath was 325 ℃, and the flow rate was 11 L/min. The mass spectrometer was operated in full-scan mode, and data were collected with a mass-to-charge ratio (m/z) between 100 and 1200 at a scanning frequency of 2 spectra per second.

The detection and quantification limits of AFB_1_ using the UPLC-MS detection method were 0.03 ng/mL and 0.1 μg/mL, respectively. Data were normalized based on the standard curves of AFB_1_ (concentrations of 1000, 500, 300, 100, 10 and 2.0 ng/mL) standard solutions. Thus, the concentration of AFB_1_ in the sample was obtained. The absolute difference between two independent measurement results obtained under repetitive conditions shall not exceed 20% of the arithmetic mean.

### 4.5. Single-Factor Experiments on Detoxification of Peanut Meal

Through preliminary experiments, it was found that the main factors affecting the removal of AFB_1_ from peanut meal by the AF01 strain were pH value, action time, temperature and inoculation dose. This study aimed to examine the effects of the above four factors on the efficiency of the AF01 strain in removing AFB_1_ from peanut meal. Five levels were set for each factor. The pH values were set to 4.0, 5.0, 6.0, 7.0 and 8.0. Solutions of 5 mol/L NaOH and 1 mol/L hydrochloric acid were used to adjust the pH of peanut meal. The pH of peanut meal was acidic, and in the pH adjustment process, it was also easy to acid reflux. Therefore, it was necessary to first over-adjust. After standing for about 0.5 h, the pH was again determined. If the pH remained stable, adjustment was halted. Otherwise, adjustment continued. The instrument used to determine the pH of peanut meal was an FE20 pH meter (Mettler Toledo Group, Zurich, Switzerland). After the pH adjustment was completed, the peanut meal was autoclaved. Sterile water and AF01 fermentation broth were then added for fermentation. The temperature was set to 5 °C, 15 °C, 30 °C, 40 °C and 50 °C. The reaction time was set to 3 h, 24 h, 48 h, 72 h and 96 h. After determining the first three factors, the effect of the inoculation dose on the solid-state fermentation degradation of AFB_1_ was explored.

The inoculation dose was set to 1%, 5%, 10%, 20% and 30%. The density of the AF01 strain in the peanut meal was measured at the beginning and end of fermentation. At the beginning of fermentation, and after 72 h of fermentation, 0.1 g of fermentation material was taken, added to 900 μL of sterile physiological saline and mixed evenly. This resulted in a 10^−2^ dilution solution. This solution was diluted sequentially with sterile physiological saline to 10^−4^, 10^−5^, 10^−6^ and 10^−7^. Then, 100 μL of the above diluent was applied on a PDA plate and incubated upside-down at 30 °C for 48 h. The colony count was observed, and the colony density (CFU/mL) was calculated.

When a certain influencing factor changed, other conditions were maintained at the natural pH of peanut meal (6.0), 5% inoculum dose and incubation for 72 h at 30 °C. After fermentation, samples from each group were poured into glass culture dishes and dried at 45 °C. Then, 10 g of dried peanut meal fermentation product was taken out and placed in a triangular flask, and 2 g of NaCl and 50 mL of extraction solution (methanol/water = 7:3, *v*/*v*) were added to it. High-speed homogenization was carried out at 10,000 r/min for 1 min using a T18 Homogenizer (IKA, Staufen, Germany). After filtering the mixture through qualitative filter paper, 15 mL of filtrate was taken out and 30 mL of deionized water was added to mix evenly. The diluted solution was filtered through microfiber filter paper, and then, 30 mL of the filtrate was injected into the aflatoxin immunoaffinity column. Finally, 1 mL of methanol was used to elute AFB_1_ from the sample, and the residual AFB_1_ in the sample was calculated using the external standard method according to the detection method described in Section 4.4. The results were expressed as the removal ratio of AFB_1_. The calculation method was as shown in Equation (2). In the control group, deionized water was used instead of the whole bacterial fermentation broth of the AF01 strain.

(2) $$\mathrm{Elimination}\;\mathrm{ratio}\;(\%)=\frac{\;\mathrm{Concentration}\;\mathrm{of}\;{\mathrm{AFB}}_{1}\;\mathrm{in}\;\mathrm{control}-\mathrm{Concentration}\;\mathrm{of}\;{\mathrm{AFB}}_{1}\;\mathrm{in}\;\mathrm{test}}{\mathrm{Concentration}\;\mathrm{of}\;{\mathrm{AFB}}_{1}\;\mathrm{in}\;\mathrm{control}}\times 100\;$$

### 4.6. Response Surface Experiment on Detoxification of Peanut Meal

Based on the single-factor experiments, Design Expert 8.0.6 software was used according to the Box–Behnken central combination design method, with three factors—action time (*A*), inoculation dose (*B*) and temperature (*C*)—as influencing factors, and with the removal ratio of AFB_1_ (*Y*) as the response value. A 3-factor and 3-level response surface optimization experiment was conducted to determine the optimal fermentation conditions for the AF01 strain to remove AFB_1_ from peanut meal. The experimental factors and levels of the response surface are shown in Table 6.

### 4.7. Increased Detoxification in Trays and Fermentation Bags

Through the response surface experiments, the optimal fermentation conditions for the removal of AFB_1_ from peanut meal by the whole bacterial fermentation broth of the AF01 strain can be determined. To explore the effectiveness of the AF01 strain in removing AFB_1_ from materials in actual production, large-scale experiments were conducted using the whole bacterial fermentation broth of the AF01 strain in shallow plates and fermentation bags. The length, width and thickness of the shallow plates were 51 cm, 36.2 cm and 4.3 cm, respectively, and they could carry 1.67 kg of material. The length and width of the fermentation bags were 55.5 cm and 49.7 cm, respectively, and they could hold up to 10 kg of material.

First, 12 kg of peanut meal and 7 kg of deionized water were separately sterilized at 121 °C and cooled naturally to room temperature. Meanwhile, according to the previously described method, 1 L of the secondary seed solution of the AF01 strain was prepared. Then, 7 kg of sterile water and 1 L of AF01 strain seed solution were added to 12 kg of sterilized peanut meal in sequence and stirred evenly. The evenly mixed materials were divided into shallow trays and fermentation bags, with each tray carrying approximately 1.67 kg of material. After loading the materials, they had to be wrapped in kraft paper to prevent bacterial contamination. Next, 5 kg of material was loaded into each fermentation bag, and the bags were sealed with a sealing machine. The shallow plates and fermentation bags were placed in a constant-temperature HPS-250 Biochemical Incubator (Harbin Donglian Electronic Technology Development Co., Harbin, China) at 29 °C for 75 h before evenly sampling from them. After fermentation, uniform samples were taken and dried to detect the residual AFB_1_ content, and the removal ratio of AFB_1_ was calculated. Sterile water was used instead of the fermentation broth of AF01 in the control group, and this experiment was repeated 3 times.

### 4.8. Determination of the Nutritional Components of Peanut Meal before and after Fermentation

After the amplification experiment’s fermentation was completed, the detoxified samples were evenly sampled and placed in a blast-drying oven at 45 °C for drying. A high-speed crusher was used to crush the samples in order to obtain fermented peanut meal. Non-inoculated peanut meal was used as the control group. The contents of phytic acid and total sugars in the peanut meal were measured, and their amino acid components were analyzed to evaluate the effect of the AF01 strain on the nutritional value of the peanut meal.

#### 4.8.1. Phytic Acid Content Determination

Sample processing: First, 1 ± 0.001 g of dried peanut meal was weighed and placed in a 150 mL triangular flask. Then, 40 mL of sodium sulfate hydrochloric acid extraction solution was added, and the mixture was shaken for 2 h to extract it. The extraction solution was centrifuged at 5000 r/min for 5 min. The supernatant was collected and diluted to 50 mL with the extraction solution. Then, it was filtered through a rapid filter paper. Next, 5 mL of filtrate was taken and added to 1 mL of NaOH solution; this was diluted to 30 mL with ultrapure water and transferred to an activated ion exchange column. The exchange column was rinsed with 15 mL of water and 15 mL of NaCl solution, and the effluent was discarded. Finally, the column was eluted with 25 mL of NaCl solution, and then the eluent was collected in a 25 mL stoppered graduated tube and filled up to the mark.

Standard curve drawing: Standard solutions with phytic acid contents of 0, 0.004, 0.01, 0.1, 0.2 and 0.5 mg were prepared; 4 mL of reaction solution was added and mixed well. After standing for 20 min, the absorbance at 500 nm was measured using a 1 cm colorimetric dish. A standard curve was drawn, with absorbance as the vertical axis and the mass of phytic acid as the horizontal axis.

Sample determination and analysis: First, 5 mL of sample eluent was transferred to a 10 mL colorimetric tube; 4 mL of reaction solution was added and mixed well. After standing for 20 min, the absorbance of the sample at 500 nm was measured using a 1 cm colorimetric dish. The phytic acid content in the sample was calculated according to Equation (3):

(3) $$X=\frac{m_{2}\times 25\times 1000}{m_{1}\times 5\times V\times 1000}\times 50$$

where *X* is the content of phytic acid in the sample (g/kg), *m*_2_ is the mass of phytic acid in 5 mL of test solution for determination (mg), 25 represents the constant volume of the eluent (mL), *m*_1_ refers to the mass of the sample (g), 5 represents the volume of the test solution used for determination (mL), *V* is the volume of the extraction solution for purification (mL) and 50 represents the constant volume of the extraction solution (mL).

#### 4.8.2. Determination of Total Sugar Content

Sample processing: First, 0.1 ± 0.001 g of the sample was weighed and poured into a 250 mL triangular flask, and then 50 mL of water and 15 mL of concentrated hydrochloric acid were added. A condensation recovery device was installed, and the mixture was placed in a 100 °C water bath for 3 h of hydrolysis. After cooling to room temperature, the filter residue was filtered and washed with distilled water. The filtrate and washing solution were combined and made up to 250 mL with water. This solution was used as the test solution for the sample for future use.

Standard curve drawing: Here, 0.1 ± 0.0001 g of dried glucose was dissolved in 100 mL of water; 0, 0.1, 0.2, 0.3, 0.4, 0.5, 0.75 and 1.0 mL glucose standard solutions were transferred to 10 mL stoppered test tubes and supplemented with distilled water to 1.0 mL. Then, 1.0 mL of 5% phenol solution and 5.0 mL of concentrated sulfuric acid were added to the glucose solution in sequence. After a 10 min static reaction, the test tube was placed in a 30 °C water bath for 20 min. An appropriate amount of reaction solution was taken, and the absorbance was measured at 490 nm. Afterward, the absorbance of the reaction solution at 490 nm was measured. A standard curve was created, with glucose concentration as the x-axis and absorbance value as the y-axis.

Sample determination and analysis: First, 0.2 mL of the test solution was transferred to a 10 mL stoppered test tube and supplemented with distilled water to 1.0 mL. The blank solution was used to zero, and the absorbance was measured. The total sugar content was calculated using a standard curve. The total sugar content in the sample was calculated as a mass fraction *w*, and the value was expressed as a percentage (%) according to Equation (4):

(4) $$w=\frac{m_{1}\times V_{1}\times 10^{-6}}{m_{2}\times V_{2}}\times 100\;$$

where *V*_1_ is the sample’s constant volume (mL), *V*_2_ is the volume of the sample measurement solution taken during colorimetric measurements (mL), *m*_1_ represents the sugar content in the sample determination solution obtained from the standard curve (μg) and *m*_2_ refers to the mass of the sample (g).

#### 4.8.3. Amino Acid Composition Determination

Sample processing: First, 0.1 ± 0.001 g of the sample was weighed and placed in a 50 mL hydrolysis tube; 20 mL of 6 mol/L hydrochloric acid solution was added, and the tube was sealed under nitrogen protection. The hydrolysis tube was subjected to hydrolysis for 22 h in a constant-temperature drying oven at 110 °C. After cooling to room temperature, the solution was made up to 50 mL with ultrapure water. Then, 2.0 mL of the fixed-volume liquid was transferred to a vacuum-drying oven and evaporated to dryness at 70 °C. The residue was washed and evaporated twice with the same volume of ultrapure water. Then, 2.0 mL of machine buffer (0.02 mol/L hydrochloric acid solution) was added for dilution and shaken well. A 0.22 μm microporous filter membrane was used for filtration of the diluent, and the filtrate was used for instrument measurement.

Standard solution preparation: First, 200 µL of amino acid mixed standard solution was piped into a 5 mL volumetric flask, diluted to volume with 0.1 mol/L hydrochloric acid solution, and used as the standard for testing on the machine. The amino acid mixed standard included 17 amino acids: aspartic acid, threonine, serine, glutamic acid, glycine, alanine, cysteine, valine, methionine, isoleucine, leucine, tyrosine, phenylalanine, lysine, histidine, arginine and proline.

Sample determination and analysis: First, 20 mL of the mixed amino acid standard working solution and 20 mL of the sample determination solution were transferred to the amino acid automatic analyzer. The amino acid concentration was calculated using the external standard method based on peak area. The detection wavelength for proline (Pro) was 440 nm, while the detection wavelength for the other amino acids was set to 570 nm. The contents of each amino acid in the standard reserve solution of mixed amino acids were calculated according to Equation (5):

(5) $$c_{j}=\frac{m_{j}}{M_{j}\times 250}\times 1000\;$$

where *c_j_* is the concentration of amino acid *j* in the standard reserve solution of mixed amino acids (μmol/L), *m_j_* is the mass of the amino acid standard substance *j* (mg), *M_j_* is the molecular weight of amino acid standard *j*, 250 refers to the fixed volume (mL) and 1000 is the conversion factor.

The amino acid contents in the sample determination solution were calculated according to Equation (6), where *c_i_* is the concentration of amino acid *i* in the sample determination solution (nmol/L), *A_i_* is the peak area of amino acid *i* in the sample determination solution, *A_s_* is the peak area of amino acid s in the amino acid standard working solution and *c_s_* is the content of amino acid s in the amino acid standard working solution (nmol/L).

(6) $$c_{i}=\frac{c_{s}}{A_{s}}\times A_{i}$$

The contents of each amino acid in the sample were calculated according to Equation (7), where *X_i_* is the content of amino acid *i* in the sample (g/100 g), *c_i_* is the concentration of amino acid *i* in the sample determination solution (nmol/mL), *F* is the dilution factor, *V* is the volume of sample hydrolysate transferred to constant volume (mL), *M* is the molar mass of amino acid *i* (g/mol), *m* is the weighing quantity (g), 10^9^ is the coefficient for converting the sample content from ng to g and 100 is the conversion coefficient.

(7) $$X_{i}=\frac{c_{i}\times F\times V\times M}{m\times 10^{9}}\times 100\;$$

### 4.9. Statistical Analysis

The strain density of AF01 in peanut meal was calculated according to the Chinese National Standard GB4789.15-2016 [48]. The density of colonies in peanut meal was expressed as CFU/g. Then, strain density data in peanut meal was log-transformed before analysis.

The elimination ratio of AFB_1_ was obtained by comparing AFB_1_ in peanut meal and fermented peanut meal according to Equation (2). The figures were drawn using GraphPad Prism software (version 9.1.1, GraphPad Software, San Diego, CA, USA). Statistical analysis was performed by one-way analysis of variance (ANOVA) followed by Duncan’s multiple range test using SPSS Statistics software (version, 29.0, SPSS Inc., Chicago, IL, USA). Different lowercase letters indicated significant differences with a confidence interval of 95%, while the same lowercase letters indicated insignificant differences (*p* > 0.05). This analysis method visualized the differences in the removal ratio of AFB_1_ under different levels of the same single factor, making the conclusions clearer.

Design Expert software (version 8.0.6, Stat-Ease, Beijing, China) was used for the design and data analysis of the response surface experiment. A quadratic regression model was established followed by a significance analysis of the model and the dependent variables involved (Table 3). Three confidence intervals were respectively set, namely 95%, 99% and 99.9%. When *p* < 0.05, the difference was significant and indicated by “*”. When *p* < 0.01, the difference was highly significant and represented by “**”. When *p* < 0.001, the difference was extremely significant and represented by “***”. This analysis method can evaluate the degree of influence of the three independent variables (time, inoculation quantity and temperature) on the dependent variable, namely removal ratio of AFB_1_. The more significant the difference, the greater the impact.

Contour lines and response surfaces were drawn using Origin software (version 2017, OriginLab, MA, USA). The steepness of the graph indicated the impact of the interaction of two factors on the removal ratio of AFB_1_.

## Figures and Tables

**Figure 2:**
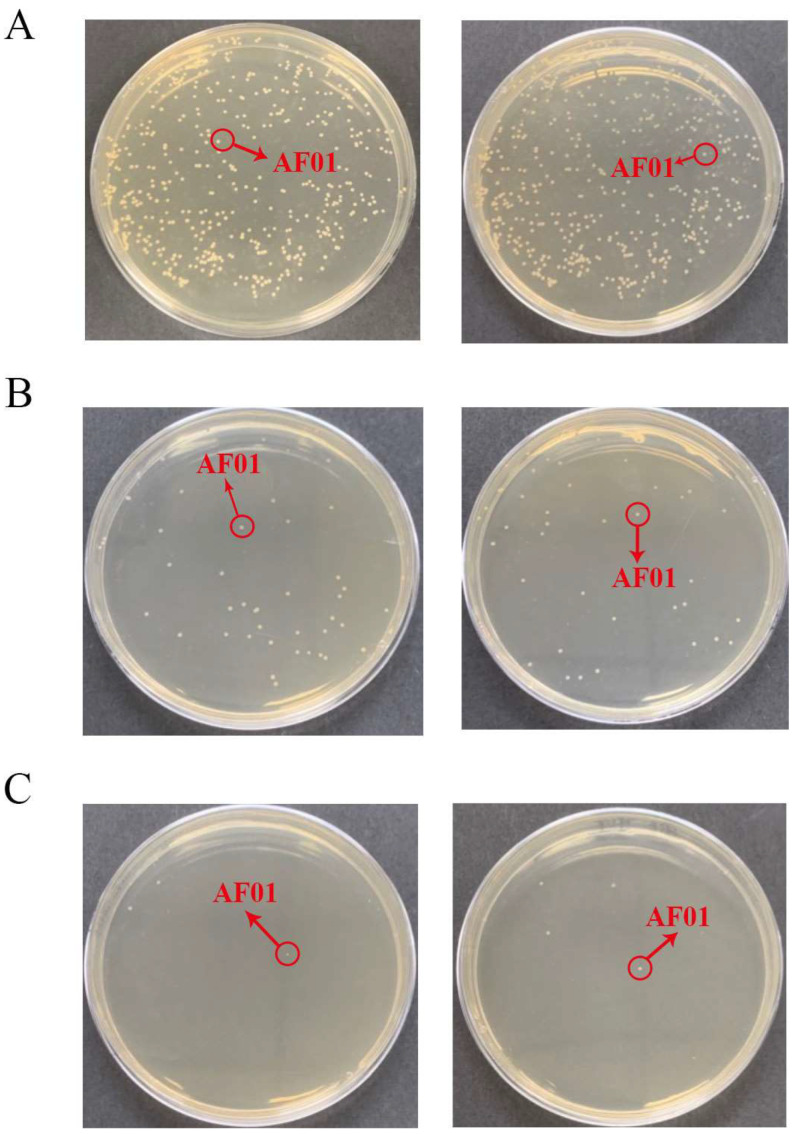
The density of the AF01 strain in peanut meal at the initial stage of the reaction: (**A**) 10^−5^ dilution; (**B**) 10^−6^ dilution; (**C**) 10^−7^ dilution. The circled and similarly shaped single colony is the strain of AF01.

**Table 1:** Changes in AF01 strain density (logCFU/g) in peanut meal treated with different inoculation doses.

Inoculation Quantity (%)	Initial Colony Density	24 h	48 h	72 h
1	6.65	8.68	9.13	9.24
5	7.34	9.51	9.72	9.81
10	7.62	9.48	9.73	9.74
20	7.88	9.38	9.11	9.49
30	8.02	8.81	9.11	9.21

**Table 2:** Design and results of Box–Behnken experiments for removal of AFB_1_ in peanut meal by AF01.

Experiment Number	*A*: Time (h)	*B*: Inoculation Quantity (%)	*C*: Temperature (°C)	Detoxification Ratio of AFB_1_ (%)
1	48	1	30	44.17
2	96	1	30	55.15
3	48	10	30	50.59
4	96	10	30	52.80
5	48	5.5	20	42.86
6	96	5.5	20	50.58
7	48	5.5	40	41.39
8	96	5.5	40	38.91
9	72	1	20	49.57
10	72	10	20	44.89
11	72	1	40	40.62
12	72	10	40	45.56
13	72	5.5	30	68.30
14	72	5.5	30	67.53
15	72	5.5	30	67.75
16	72	5.5	30	70.53
17	72	5.5	30	67.89

**Table 3:** ANOVA of the quadratic regression model.

Variance Source	Sum of Squares	Degrees of Freedom	Mean Square	*F* Value	*p*-Value	Significance
Model	1989.38	9	221.04	82.92	<0.0001	***
*A*	42.46	1	42.46	15.93	0.0052	**
*B*	2.34	1	2.34	0.88	0.3796	–
*C*	57.35	1	57.35	21.52	0.0024	**
*AB*	19.23	1	19.23	7.21	0.0313	*
*AC*	26.01	1	26.01	9.76	0.0168	*
*BC*	23.14	1	23.14	8.68	0.0215	*
*A* ^2^	398.08	1	398.08	149.34	<0.0001	***
*B* ^2^	269.36	1	269.36	101.05	<0.0001	***
*C* ^2^	978.03	1	978.03	366.91	<0.0001	***
Residual	18.66	7	2.67			
Lack of fit	12.67	3	4.22	0.50	0.1712	Not significant
Pure error	5.99	4	1.50			
Core total	2008.04	16				

Note: ‘–’ indicates no significant difference; ‘*’ (*p* < 0.05) indicates significant differences; ‘**’ (*p* < 0.01) indicates highly significant differences; ‘***’ (*p* < 0.001) indicates extremely significant differences.

**Table 4:** Nutritional composition of peanut meal before and after fermentation.

	Total Sugar Content (%)	Total Amount of Amino Acids (%)	Phytic Acid Content (%)
Peanut meal in the control group	35.27 ± 0.37	42.66 ± 0.25	1.47 ± 0.10
Fermented peanut meal	39.37 ± 0.64	47.05 ± 0.28	0.68 ± 0.12
% Change	+4.10%	+4.39%	−0.79%

**Table 5:** Amino acid contents of peanut meal before and after fermentation.

Amino Acid Name	Abbreviation	Amino Acid Content in Control Group (%)	Amino Acid Content in Experimental Group (%)	% Increase
Aspartate	Asp	5.39	6.01	0.62
Threonine	Thr	1.37	1.46	0.09
Serine	Ser	2.34	2.62	0.28
Glutamate	Glu	9.04	9.94	0.90
Glycine	Gly	2.68	2.93	0.25
Alanine	Ala	1.95	2.16	0.21
Cysteine	Cys	0.42	0.47	0.05
Valine	Val	1.87	2.11	0.24
Methionine	Met	0.35	0.36	0.01
Isoleucine	Ile	1.49	1.69	0.20
Leucine	Leu	3.16	3.52	0.36
Tyrosine	Tyr	1.53	1.66	0.13
Phenylalanine	Phe	2.41	2.62	0.21
Lysine	Lys	1.5	1.71	0.21
Histidine	His	1.03	1.14	0.11
Arginine	Arg	4.97	5.19	0.22
Proline	Pro	1.16	1.46	0.30

**Table 6:** Optimization of response surface experiment design factors and levels for aflatoxin B_1_ removal.

Level	*A*: Action Time (h)	*B*: Inoculation Quantity (%)	*C*: Temperature (°C)
−1	48	1	20
0	72	5.5	30
1	96	10	40

## Data Availability

Data are contained within the article and Supplementary Materials.

## Supplementary Materials

The following supporting information can be downloaded at: https://www.mdpi.com/article/10.3390/toxins16070305/s1, Figure S1: shallow-plate fermentation application experiment: (A) before fermentation; (B) during the fermentation process; (C) after fermentation.; Figure S2: fermentation-bag application experiment: (A) before fermentation; (B) during the fermentation process; (C) after fermentation; Figure S3: standard curve for phytic acid determination; Figure S4: standard curve of glucose concentration; Figure S5: peak plot of amino acid standards.
