# Supplementary material for: Bioremediation of Aflatoxin B1 by Meyerozyma guilliermondii AF01 in Peanut Meal via Solid-State Fermentation
Source: Toxins (Basel). 2024 Jul 4;16(7):305. doi: 10.3390/toxins16070305 (PMC11280932; doi:10.3390/toxins16070305)
Supplement: Supplementary file 1 [file toxins-16-00305-s001.zip › toxins-3055667-supplementary.pdf]

Supplementary Materials: Bioremediation of Aflatoxin B_1 by *Meyerozyma guilliermondii* AF01 in Peanut Meal via Solid-State Fermentation

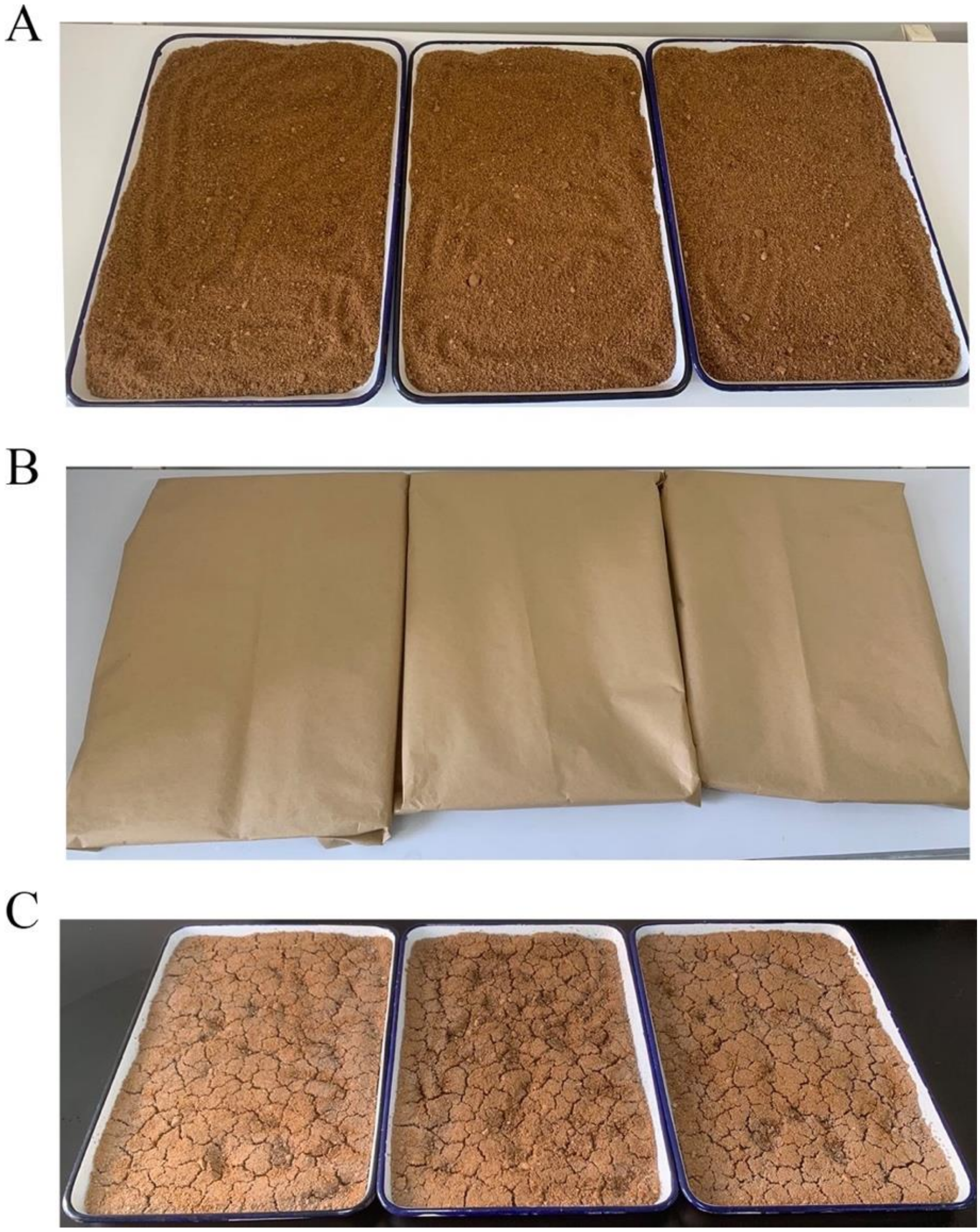

Figure S1. Shallow-plate fermentation application experiment: (A) before fermentation; (B) during the fermentation process; (C) after fermentation.

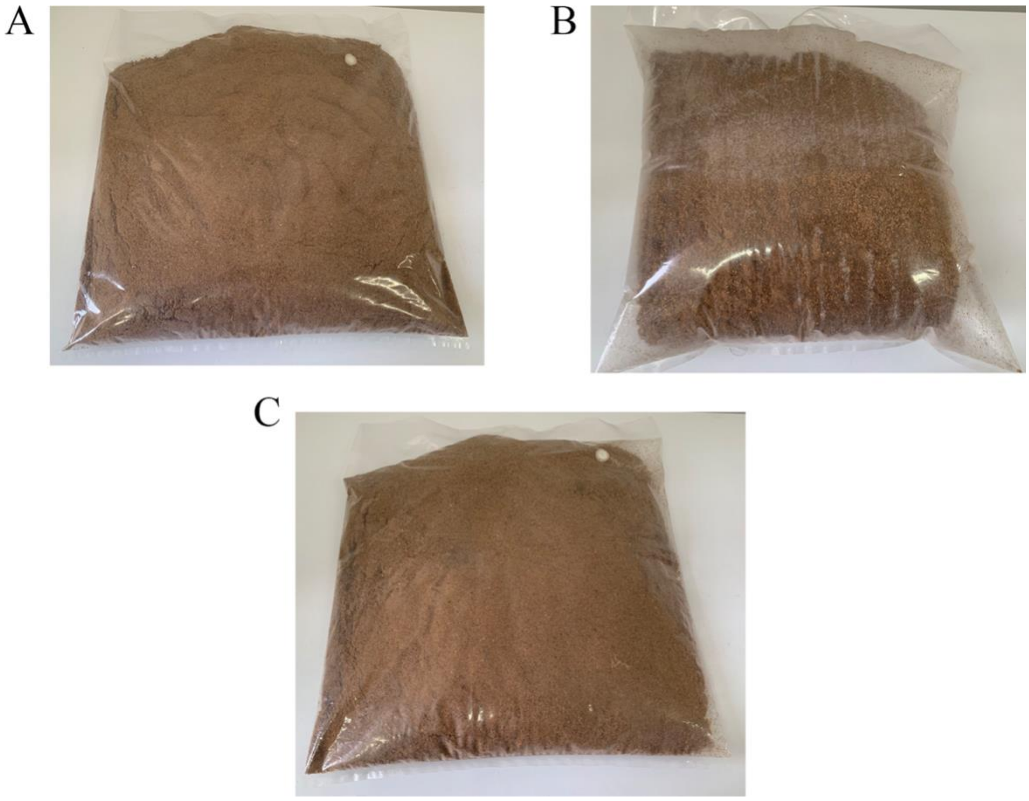

Figure S2. Fermentation bag application experiment: (A) before fermentation; (B) during the fermentation process; (C) after fermentation.

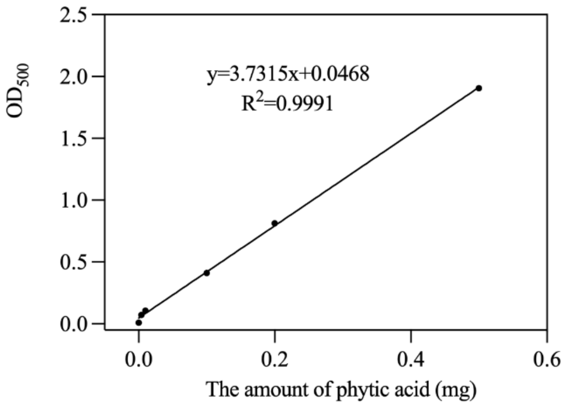

Figure S3. Standard curve for phytic acid determination.

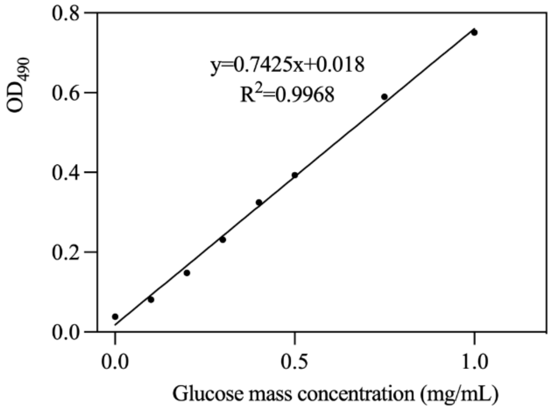

Figure S4. Standard curve of glucose concentration.

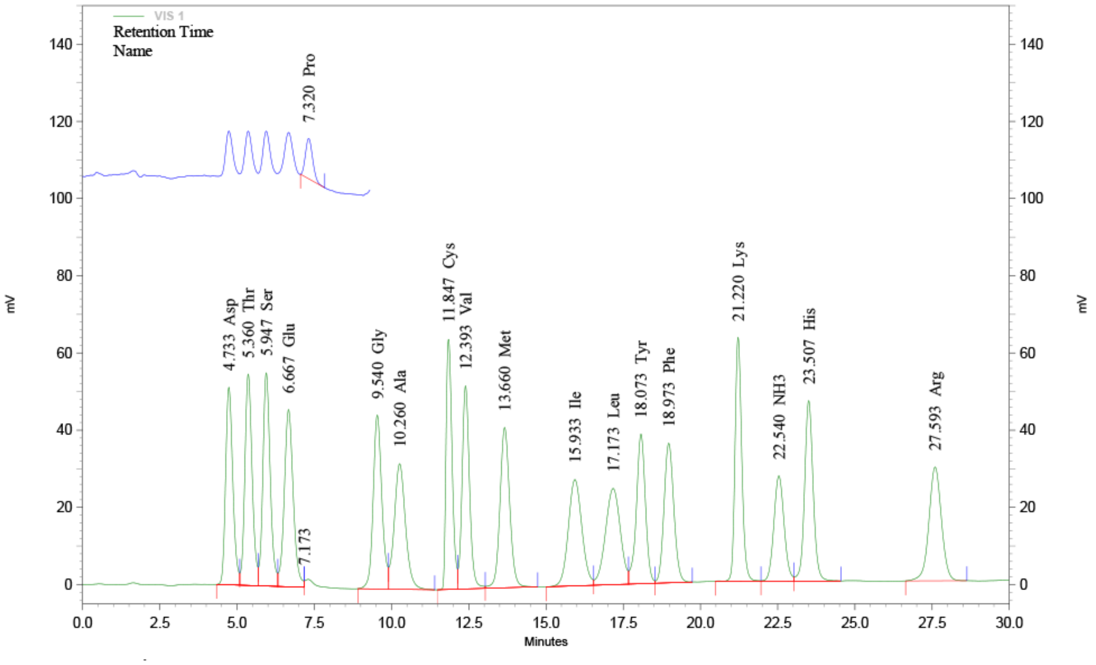

Figure S5. Peak plot of amino acid standards.
